# A SOCS1/3 Antagonist Peptide Protects Mice Against Lethal Infection with Influenza A Virus

**DOI:** 10.3389/fimmu.2015.00574

**Published:** 2015-11-11

**Authors:** Chulbul M. Ahmed, Rea Dabelic, Simone Kennedy Bedoya, Joseph Larkin, Howard M. Johnson

**Affiliations:** ^1^Department of Microbiology and Cell Science, University of Florida, Gainesville, FL, USA

**Keywords:** antivirals, SOCS antagonist, influenza A PR/8 virus, innate immunity, SOCS

## Abstract

We have developed an antagonist to suppressor of cytokine signaling 1 (SOCS1), pJAK2(1001–1013), which corresponds to the activation loop of the Janus kinase JAK2, which is the binding site for the kinase inhibitory region (KIR) of SOCS1. Internalized pJAK2(1001–1013) inhibits SOCS1 and SOCS3. SOCS1 has been shown to be an influenza virus-induced virulence factor that enhances infection of cells. The antagonist was protective in cell culture and in influenza virus PR8 lethally infected C57BL/6 mice. The SOCS antagonist also prevented adverse morbidity as assessed by parameters, such as weight loss and drop in body temperature, and showed potent induction of both the cellular and humoral immune responses to the influenza virus candidate universal antigen matrix protein 2 (M2e). The SOCS antagonist, thus, protected mice against lethal influenza virus infection and possessed potent adjuvancy against the M2e candidate influenza virus universal vaccine antigen.

## Introduction

The process of activation of cells by cytokines, such as the interferons (IFNs), in response to viral infections also activates an inducible cytokine regulatory system called suppressors of cytokine signaling (SOCS). There are currently eight known members of the SOCS family, SOCS1 to SOCS7 and cytokine-inducible Src homology 2 (SH2) protein (1). SOCS1 plays a central role in regulation of IFN signaling via inhibition of JAK/STAT signaling. The N terminus of SOCS1 contains an SH2 domain, and N-terminal to it is an extended SH2 sequence adjacent to a kinase inhibitory region (KIR). These regions or domains of SOCS1 bind to the activation and catalytic regions of JAK2 and block its function. The C terminus of SOCS1 contains a domain called the SOCS box, which is involved in proteasomal degradation of JAK2. We have shown that the KIR sequence of SOCS1 binds to a peptide corresponding to the phosphorylated activation loop of JAK2, pJAK2(1001–1013), and demonstrated that internalized pJAK2(1001–1013) blocked SOCS1 activity in cells (2, 3). Specifically, pJAK2(1001–1013) enhances suboptimal IFN activity, blocks SOCS1-induced inhibition of STAT3 activation, enhances IFNγ activation site (GAS) promoter activity, and enhances antigen-specific proliferation. It is, thus, a potential antiviral therapeutic.

There is a dynamic interaction between the JAK kinases that mediate the antiviral effects of IFNs and the IFN-induced SOCS molecules (such as SOCS1), which prevents unregulated IFN activity (4). Along with the well-known induction of IFN in cells, there is also the constitutive or endogenous presence of IFNβ, which interacts in complex ways with other IFNs in the induction of antiviral states and other functions (5, 6). Specifically, endogenous IFNβ is the key to enhancing the biological activity of induced and added IFNαs and IFNγ (5, 6). SOCS1 antagonist administered intracellularly enhances endogenous IFNβ levels by blocking the activity of both basal and induced SOCS1 (3). Thus, SOCS1 antagonist enhances IFN activity.

We have previously shown that pJAK2(1001–1013) antagonized the IFN inhibitory effect of SOCS1 in herpes simplex virus 1 (HSV-1)-infected keratinocytes (7). More recently, we showed that pJAK2(1001–1013) inhibited the replication of vaccinia virus and encephalomyocarditis virus in cell culture via inhibition of SOCS1 (3). In the same study, pJAK2(1001–1013) protected mice against lethal vaccinia and encephalomyocarditis virus infection.

It was recently shown that influenza A virus PR8 H1N1 infection of a human alveolar epithelial cell line induced early expression of SOCS1 and later expression of SOCS3 (8). SOCS expression correlated with virus refractiveness to type I IFN therapy of infected cells. Similar results were obtained by others where it was shown that the virus induction of SOCS1 and SOCS3 occurred via a retinoic acid-inducible gene 1/type 1 IFN receptor IFNAR1-dependent pathway (9).

Although there is interest in various innate response mechanisms to influenza virus infection, including the IFN response, there is no concerted or serious effort to use IFNs as therapeutics, perhaps because there is no obvious convenient use of these proteins on a large scale. Currently, virus neuraminidase inhibitors, particularly oseltamivir (Tamiflu), are the most widely used influenza therapeutics where their therapeutic efficacy has been variable, with the demonstration of oseltamivir resistance associated with neuraminidase mutations (10). Along with the gastrointestinal side effects, neuraminidase inhibitors have also been associated with neuropsychiatric effects (11).

In this report, we determined the ability of the SOCS1 antagonist to protect mice against a lethal dose of influenza A/PR8 (H1N1) infection. We further determined if protective effects were accompanied by an adjuvant effect in enhancing the innate and adaptive immune responses to influenza virus infection. SOCS proteins function intracellularly only in cells that produce them and are thus not secreted to exert intercellular effects. Accordingly, we have established that the SOCS1 antagonist has to be internalized in order to interact with and inhibit both constitutive and induced SOCS1. SOCS1 antagonist pJAK2(1001–1013) was, thus, synthesized with an attached lipophilic (lipo) palmitate for cell penetration (3) in studies reported here.

## Materials and Methods

### Peptide Synthesis

SOCS1/3 antagonist, its control peptide, as well as influenza M2e peptide were synthesized using fluorenylmethyloxycarbonyl chemistry, as previously described (12). A lipophilic group (palmitoyl-lysine) for cell penetration was added to the N-terminus as a last step of synthesis, using a semi-automated protocol. The sequence of SOCS1/3 antagonist peptide is, LPQDKEpYYKVKEP (pJAK2). The control peptide that has the tyrosines replaced by alanines and lacks biological activity has the following sequence, LPQDKEAAKVKEP (JAK2A). Influenza A virus M2e peptide had the following sequence, MSLLTEVETPTRNGWECRCSDSSD. Peptides were dissolved in DMSO followed by dilution in PBS.

### Cell Culture and Antiviral Assays

MDCK cells were grown in DMEM with 10% FBS and antibiotics. These cells were infected with influenza A/PR8 virus (10^3^ TCID_50_/ml), and incubated for 1 h at 35°C. The media was removed and replaced with DMEM with FBS and the cells were incubated for an additional 24 h at 35°C. The cells were stained with crystal violet and absorbance was measured.

### Mice

All animal protocols were approved by the Institutional Animal Care and Use Committee at the University of Florida. Female C57BL/6 mice (6–8 weeks old) were purchased from The Jackson Laboratory (Bar Harbor, ME, USA). Peptides dissolved in PBS in a volume of 100 μl were administered i.p. Mouse adapted influenza A/PR8 virus (10× LD_50_ pfu) was administered intranasally taken in a volume of 10 μl, with 5 μl delivered in each of the nostrils of a lightly anesthetized mouse. Following infection, mice were observed daily for signs of disease, such as lethargy, ruffled hair, weight loss, and eye secretions. Moribund mice were euthanized and counted as dead.

### Measurement of Influenza A Virus-Specific Cellular and Humoral Immune Responses

Spleens from naive or mice immunized intraperitoneally with M2e peptide (50 μg) in the presence of 200 μg of pJAK2(1001–1013), or control peptide (scram) were harvested after 4 weeks and homogenized to single-cell suspension. Splenocytes (10^5^ cells/well) were incubated with medium alone or medium containing M2e peptide (50 μg) at 37°C for 72 h. CellTiter Aqueous One Cell Proliferation Assay reagent (Promega) was added and the absorbance was measured.

Blood was drawn from naïve mice or those immunized with M2e peptide in the presence of pJAK2 or control peptide after 2 or 3 weeks. Microtiter plates coated with M2e peptide (10 μg/well) were blocked with PBS containing 5% FBS for 2 h at room temperature. Mouse serum was serially diluted (0.1 ml) in PBS containing 0.1% Tween 20 (wash buffer). Plates were incubated for 2 h at room temperature and washed three times with wash buffer. Peroxidase-conjugated goat anti-mouse IgG (Santa Cruz Biotechnology), diluted in a volume of 0.1 ml, was added to each well, incubated for 1 h, and washed five times with wash buffer. *o*-Phenylenediamine (OPD) (0.1 ml) was added and incubated for 15 min. The reaction was stopped by addition of 50 μl 3 N HCl. The OD at 490 nm was determined using a microtiter plate reader.

### Alexa Fluor 647 Labeling and Detection of Cell Penetration

Alexa Fluor 647 was conjugated to lipo-pJAK2(1001–1013) according to the manufacturer’s (Invitrogen) instructions. Mice were injected i.p. with 15 μg Alexa Fluor 647-labeled lipo-pJAK2(1010–1013) or an equivalent amount of Alexa Fluor alone. Two hours later, different organs were harvested and viewed under a Xenogen IVIS fluorescence imager. Cells isolated from spleen, lymph nodes, and peritoneal cavity were labeled with antibodies to CD4^+^, CD8^+^, B220^+^, and CD11b^+^ immune cells and analyzed by flow cytometry.

### Statistical Analysis

All experimental data were analyzed for statistical significance by Student’s *t*-test. For mice studies, Kaplan–Meier survival curve and log-rank test were used. GraphPad Prism from GraphPad Software (San Diego, CA, USA) was used for statistical analysis.

## Results

To determine if lipo-pJAK2(1001–1013) possessed anti-influenza virus activity in cell culture, we first incubated the antagonist with MDCK cells for 18 h. As a control, we similarly treated cells with the SOCS1 antagonist control peptide where the tyrosines at residues 1007 and 1008 of the peptide were replaced with alanines. Phosphorylated tyrosine 1007 has been shown to play an essential role in activated JAK2(pJAK2) enzymatic activity (2). We have shown that wild-type pJAK2(1001–1013) interacted with KIR of SOCS1 much more effectively than the alanine-substituted JAK2(1001–1013)2A and possessed antiviral activity against vaccinia virus, while the substituted peptide failed to antagonize SOCS1 in cell cultures and in mice infected with the virus (3).

**Figure 1:**
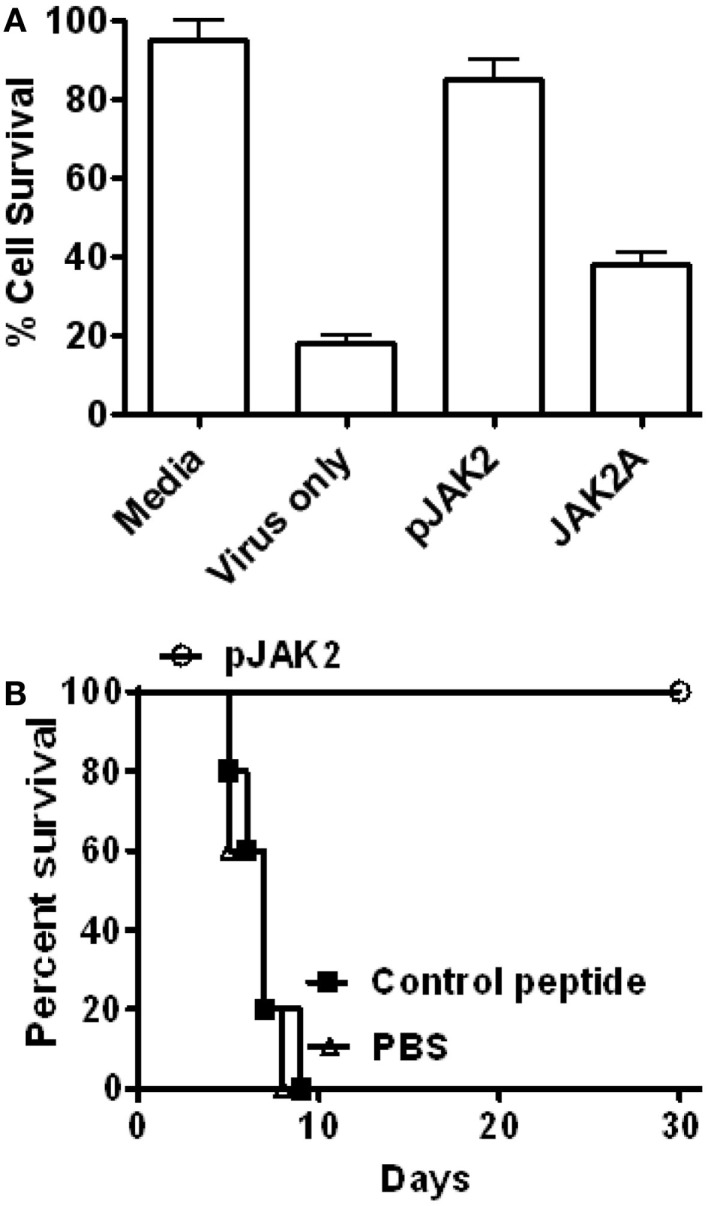
**SOCS antagonist protects mice against influenza A virus infection**. **(A)** MDCK cells were infected with influenza A virus in the presence of SOCS1 antagonist or its control peptide. After 24 h of infection, cells were stained with crystal violet and the cell survival was measured. The significance of differences was *p* = 0.001, *p* = 0.006, and *p* = 0.001 for virus only, pJAK2 and control peptide, respectively, versus media only. **(B)** Mice (C57BL/6, *n* = 5) were infected intranasally with 10× LD_50_ of influenza A PR/8 (H1N1) virus. Starting with day 1, PBS, or 200 μg each of SOCS antagonist, or the alanine-substituted antagonist control peptide were injected i.p. daily for 1 week. Palmitate was attached to all peptides for cell penetration. Survival of mice was followed. *p* Value of <0.01 was observed for the SOCS antagonist versus the control peptide. For more details on the peptides, see Section “Materials and Methods.”

As shown in Figure 1A, the SOCS antagonist but not the control peptide blocked influenza PR8 virus replication in the MDCK cells as reflected by lack of virus induced cytopathogenic effect (CPE) in antagonist-treated cells. Specifically, antagonist-treated cells infected with a 10^3^ tissue culture infectivity doses of virus per milliliter (10^3^ TCID_50_/ml) showed 85% survival, similar to that of untreated cells, while untreated virus-infected cells showed 18% survival. Alanine-substituted control peptide treated cells showed 40% survival. These tissue culture results, thus, suggest that the SOCS1 antagonist could potentially show antiviral activity against influenza virus infection in mice.

As shown in Figure 1B, the SOCS1 antagonist completely protected C57BL/6 mice from a 10× LD_50_ dose of influenza virus, while the control peptide treated mice all died by day 9, similar to PBS-treated mice. The surviving mice did not show any signs of illness for four additional weeks until the completion of the study. Treatment involved IP injection of 200 μg peptide daily for 1 week, beginning 1 day post-intranasal infection. Thus, the SOCS1 antagonist possessed potent therapeutic efficacy against mice infected with a lethal dose of influenza virus.

**Figure 2:**
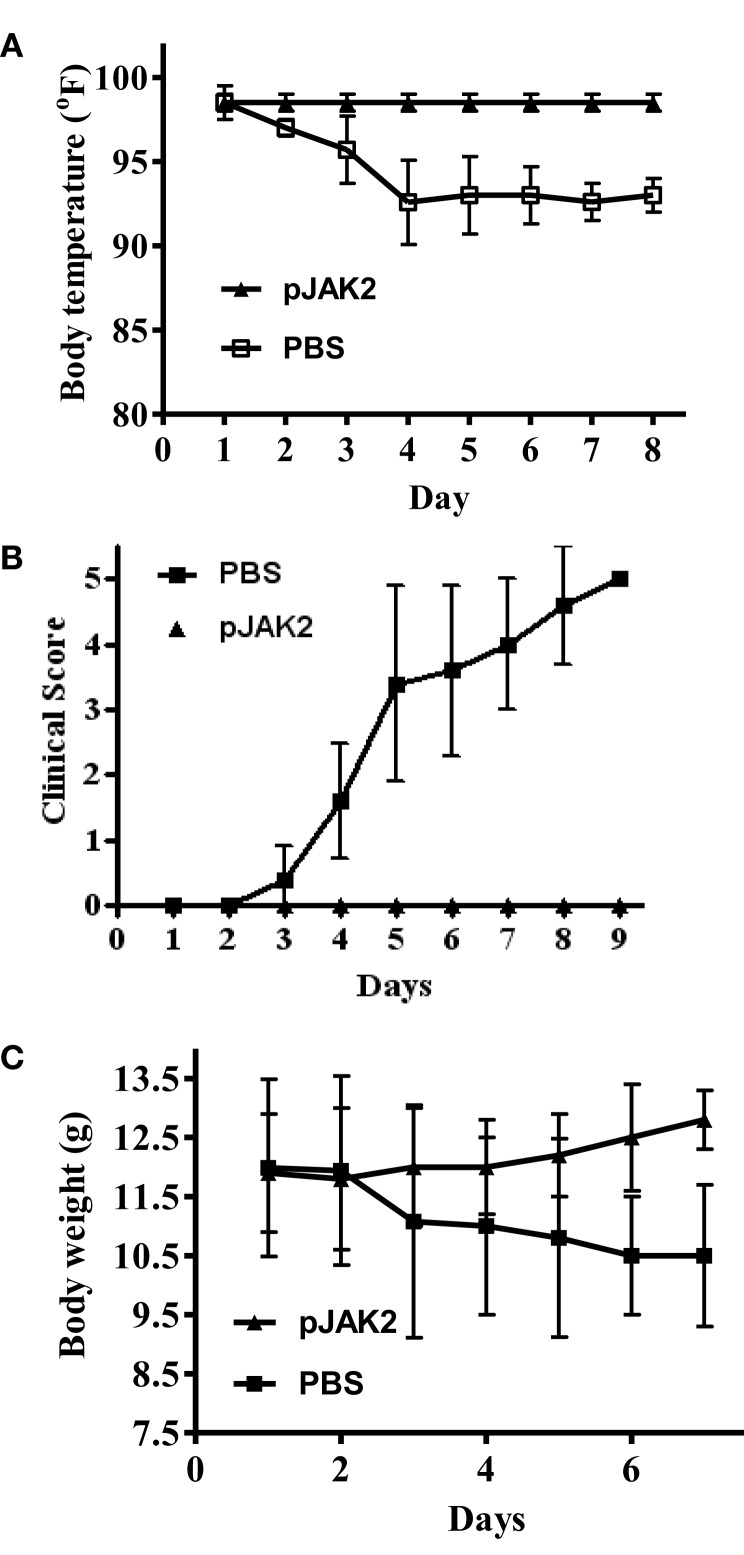
**SOCS antagonist protects against the onset of symptoms in influenza A virus infection**. C57BL/6 mice were infected intranasally as in Figure 1 on day 0 with 10× LD50 of influenza A PR/8 virus. Starting with day 1, daily injections of PBS or pJAK2 (200 μg) were given i.p. All the PBS-injected mice were dead by day 9, while the pJAK2-treated mice survived the virus challenge. Changes in body temperature in Fahrenheit are indicated in **(A)**. The *p* value between pJAK2 peptide versus PBS was 0.001. Clinical score (see text) is shown in **(B)**, where *p* = 0.002 between pJAK2- versus PBS-treated mice. Body weight in grams is recorded in **(C)**. The significance of difference between pJAK2 and PBS is *p* = 0.002.

In order to obtain some insight into the ability of the SOCS1 antagonist to protect mice against infection, we focused on several parameters that reflect the degree of morbidity. SOCS1 antagonist-treated mice showed no drop in body temperature, while control mice experienced approximately a 5% drop in temperature at days 4–8 post-infection (Figure 2A). We assessed clinical scores on the well-being of the mice by the following scale: 1, ruffled hair, hunched posture; 2, 1+ lethargic; 3, weight loss; 4, moribund; and 5, death. As shown in Figure 2B, control mice showed increasing clinical scores over time, while the clinical scores of SOCS1 antagonist-treated mice stayed the same. Consistent with all of this, control mice experienced a dramatic loss of weight due to infection, approaching 15% by day 7 (Figure 2C), while antagonist-treated mice gained weight. The antagonist, thus, protected mice against the symptoms associated with influenza virus infection.

**Figure 3:**
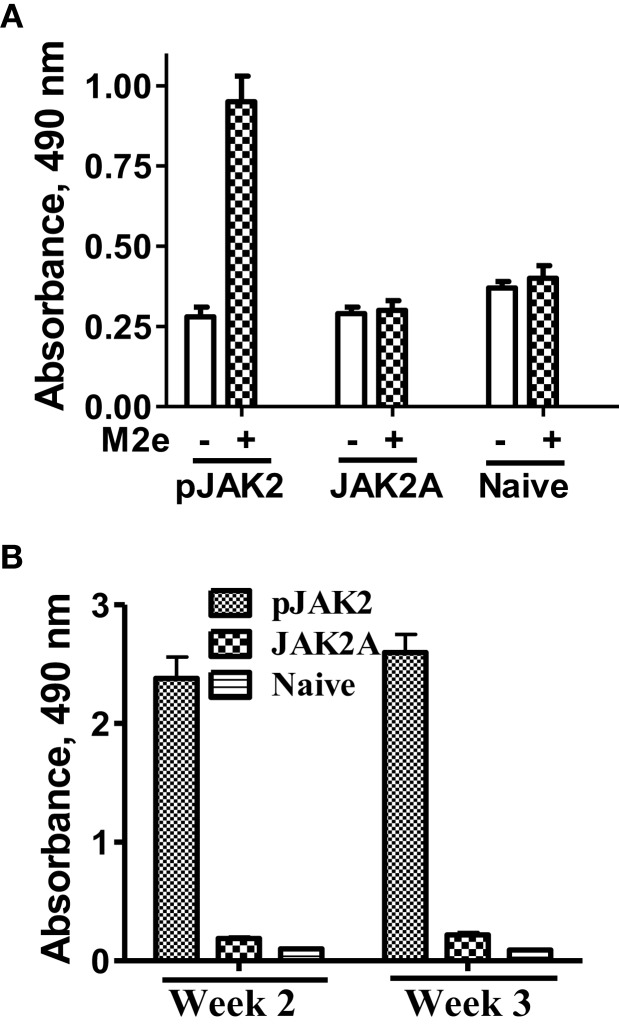
**pJAK2(1001–1013) exhibits adjuvant properties at both cellular and humoral levels**. **(A)** Spleens were harvested from C57BL/6 mice (*n* = 3) 4 weeks after immunization with M2e as an antigen and treatment with pJAK2 or the control peptide. Naïve mice were included as a control. Splenocytes (5 × 10^5^ cells per well) in microtiter plates were incubated with or without M2e (50 μg/ml) for 72 h. CellTiter Aqueous One Cell Proliferation Assay reagent was added and absorbance was read. *p* Value of <0.01 was observed between pJAK2-treated and naïve mice. **(B)** Mice were immunized using M2e as an antigen in the presence of pJAK2 peptide or the control peptide. After 2 and 3 weeks, blood was drawn from the mice and measured for the presence of M2e-specific antibodies in an ELISA format. The values represent average with SD. *p* Value of <0.05 was obtained for the SOCS antagonist versus the control peptide.

In addition to protection in terms of morbidity and mortality, we were interested in determining if the SOCS1 antagonist possessed adjuvant effects against the influenza virus. We focused on the conserved extracellular domain of the matrix protein 2 (M2e), which is highly conserved across the various human influenza A virus strains. We synthesized the 24-amino acid M2e consensus sequence and injected mice IP with 200 μg M2e along with 200 μg SOCS1 antagonist or antagonist control peptide. Spleens were removed after 4 weeks and the cells were incubated with M2e (50 μg/ml) for 72 h and cellular proliferation was assessed via the Cell Titer Aqueous One Cell proliferation assay (Promega, Madison, WI, USA). As shown in Figure 3A, proliferation was observed only in mice that were injected with M2e and the SOCS1 antagonist. The SOCS1 antagonist control-treated mice showed a profile similar to that of naïve mice. Serum from the same mice showed an antibody response to M2e only when the mice were also injected with SOCS1 antagonist (Figure 3B). The SOCS1 antagonist, thus, possessed adjuvancy at the cellular and humoral level of immune response to the M2e universal influenza virus antigen.

**Figure 4:**
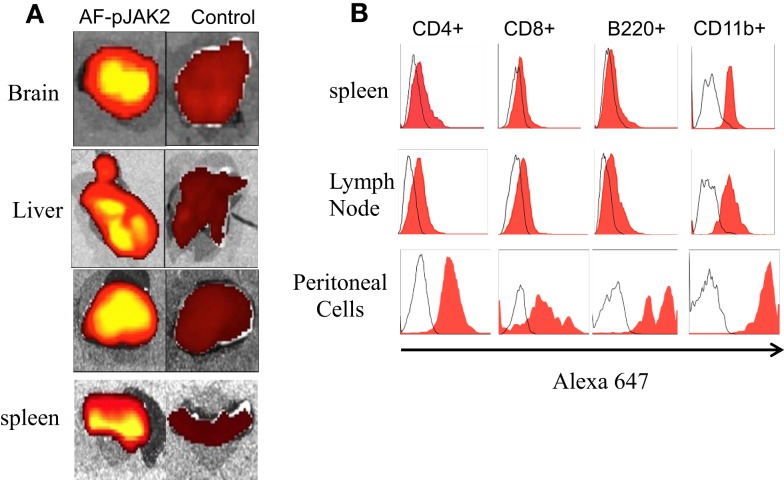
**Tissue and immune cell localization of Alexa Fluor 647 conjugated lipo-pJAK2 (AF-pJAK2) peptide**. Lipo-pJak2 was conjugated to the fluorochrome Alexa 647 (Invitrogen). AF-pJAK2 or unconjugated fluorochrome was injected into C57BL/6 mice i.p., followed by sacrifice of the mice 2 h later. Brain, liver, kidney, spleen, lymph node, and peritoneal cells were subsequently isolated. **(A)** AF-pJAK2 incorporation into the brain, liver, kidney, and spleen of mice receiving AF-pJAK2 i.p (left) in contrast to mice receiving unconjugated Alexa 647 (FC, right) using a Xenogen IVIS Fluorescence Imager. **(B)** AF-pJAK2 is incorporated into immune cells. Histograms showing CD4^+^, CD8^+^, B220^+^, and CD11b^+^ leukocytes present within the spleen, lymph node, and peritoneal cavity of AF-pJAK2-injected mice that stained positive for AF-647 (red) when compared to leukocytes isolated from mice receiving unconjugated AF-647 i.p. (uncolored histogram). Data are representative of two independent experiments with four mice in each group.

Using Alexa Fluor 647 conjugated-lipo-pJAK2(1001–1013) (AF-pJAK2), we assessed organ and immune cell presence of the antagonist. Specifically, C57BL/6 mice were injected IP with 200 μg AF-pJAK2 and 2 h later, organs and tissues were harvested and analyzed for the presence of AF-pJAK2 in brain, liver, kidney, and spleen (Figure 4A). Control organs from mice-injected with Alexa 647 alone showed relative absence of the fluorochrome. Cell sorting showed the presence of AF-pJAK2 in CD4^+^, CD8^+^, B220^+^, and CD11b^+^ cells of the spleen, lymph node, and antigen-presenting cells relative to the Alexa 647 control (Figure 4B). Thus, lipo-pJAK2(1001–1013) showed wide organ and immune cell presence in treated mice.

## Discussion

The absolute critical importance of SOCS in regulation of cytokines, such as the IFNs, is underscored by the demonstration that homozygous knockout of the SOCS1 gene in mice results in lethal neonatal inflammatory disease due in large part to unregulated IFNγ activity (13). Thus, most JAK/STAT signaling that is related to innate and adaptive immunity also temporally activates genes, such as SOCS1, to mitigate against prolonged expression or overexpression of cytokines that might result in inflammatory damage to the individual. Both SOCS1 and SOCS3 appear to play important roles in influenza virus infection at the cellular level. Specifically, it has recently been shown that influenza A/PR8 infection of a human alveolar epithelial cell line induced early expression of SOCS1 and later expression of SOCS3 (8, 9). Although SOCS expression correlated with virus refractiveness to type I IFN therapy of infected cells, a definitive causal relationship was not shown. Additionally, there were no animal model studies done that actually showed that induction of SOCS played a role in influenza virus pathogenesis.

A well-recognized but not fully understood aspect of IFN function in cells is that most cells constitutively produce low levels of intracellular IFNβ that have been shown to play a role in induction of an antiviral state in cells treated with type I and type II IFNs (5, 6). pJAK2(1001–1013) increased the level of intracellular IFNβ in cells in which it induced the antiviral state. In addition, pJAK2(1001–1013) enhanced STAT1α transcription factor activation and synergized with IFNγ mimetic at the level of transcription to induce gene activation through the GAS promoter (3). This provides insight into the mechanistic effects of the SOCS antagonist at the level of signal transduction and gene activation.

In addition to SOCS, regulatory T cells are an important regulatory arm of the immune system (14, 15). We and others have shown that there is cross-talk between these two regulatory arms (15). Importantly, we showed that there was a hierarchy between the two systems, where SOCS1 is required for a functional peripheral regulatory T cell system. This suggests that the SOCS1/3 antagonist controls regulatory T cells via its regulation of the SOCS system. Our SOCS antagonist should, thus, have potential for direct treatment of influenza virus infections, but also should play a role in enhancement of immunogenicity of universal influenza virus antigens such as the M2e polypeptide.

Given that our SOCS1/3 antagonist is effective against a broad group of viruses, such as HSV-1, vaccinia virus, EMC virus, and here against influenza virus, we feel that it should be a candidate for treatment of virus infections in general, including that of the Ebola virus and members of the Flaviviridae family. It has recently been shown, for example, that Ebola virus infection of the human HEK293 cell line results in induction of SOCS1 (16). Two members of the Flaviviridae family of viruses, dengue virus and West Nile virus, similarly induce SOCS, specifically SOCS1 and SOCS3, and this has been considered as potentially inhibiting the IFN response in these infections (17).

A recent study showed that the SOCS1 antagonist enhanced antigen presentation of human dendritic cells for increased cytotoxic T cell response against human gastric cancer cells (18). This broadens the potential use of pJAK2(1001–1013) beyond the role of an antiviral, and also as an enhancer of the immune system in host defense against cancer, as we have shown previously (19). In a general sense, it would suggest that SOCS, such as SOCS1, should be considered in circumstances where increased immune activity is desirable against infectious as well as non-infectious diseases such as cancer.

## Conflict of Interest Statement

The authors declare that the research was conducted in the absence of any commercial or financial relationships that could be construed as a potential conflict of interest.
